# Genome-wide linkage mapping of root system architecture-related traits in common wheat (*Triticum aestivum* L.)

**DOI:** 10.3389/fpls.2023.1274392

**Published:** 2023-10-13

**Authors:** Yirong Jin, Yamei Wang, Jindong Liu, Fuyan Wang, Xiaodong Qiu, Peng Liu

**Affiliations:** ^1^ Wheat Research Institute, Dezhou Academy of Agricultural Sciences, Dezhou, China; ^2^ School of Agriculture, Sun Yat-sen University, Shenzhen, China; ^3^ Institute of Crop Sciences, Chinese Academy of Agricultural Sciences, Beijing, China; ^4^ Department of Science and Technology of Shandong Province, Jinan, China

**Keywords:** bread wheat, marker-assisted selection, quantitative trait locus (QTL), root system architecture (RSA), single-nucleotide polymorphism (SNP)

## Abstract

Identifying loci for root system architecture (RSA) traits and developing available markers are crucial for wheat breeding. In this study, RSA-related traits, including total root length (TRL), total root area (TRA), and number of root tips (NRT), were evaluated in the Doumai/Shi4185 recombinant inbred line (RIL) population under hydroponics. In addition, both the RILs and parents were genotyped using the wheat 90K single-nucleotide polymorphism (SNP) array. In total, two quantitative trait loci (QTLs) each for TRL (*QTRL.caas-4A.1* and *QTRL.caas-4A.2*), TRA (*QTRA.caas-4A* and *QTRA.caas-4D*), and NRT (*QNRT.caas-5B* and *QNRT.caas-5D*) were identified and each explaining 5.94%–9.47%, 6.85%–7.10%, and 5.91%–10.16% phenotypic variances, respectively. Among these, *QTRL.caas-4A.1* and *QTRA.caas-4A* overlapped with previous reports, while *QTRL.caas-4A.2*, *QTRA.caas-4D*, *QNRT.caas-5B*, and *QNRT.caas-5D* were novel. The favorable alleles of *QTRL.caas-4A.1*, *QTRA.caas-4A*, and *QTRA.caas-5B* were contributed by Doumai, whereas the favorable alleles of *QTRL.caas-4A.2*, *QTRA.caas-4D*, and *QTRA.caas-5D* originated from Shi 4185. Additionally, two competitive allele-specific PCR (KASP) markers, *Kasp_4A_RL* (*QTRA.caas-4A*) and *Kasp_5D_RT* (*QNRT.caas-5D*), were developed and validated in 165 wheat accessions. This study provides new loci and available KASP markers, accelerating wheat breeding for higher yields.

## Introduction

Wheat production is influenced seriously by abiotic stresses. Breeding higher-yielding and more stable accessions under abiotic stress is a crucial objective in modern wheat breeding (Bai et al., 2013; Alahmad et al., 2019; Adeleke et al., 2020). Root system architecture (RSA) traits, which contribute to the shape of the root system, are pivotal for wheat agronomic performance and play vital roles in plant development (Kabir et al., 2015; Li et al., 2015; Kulkarni et al., 2017; Alemu et al., 2021). Optimizing RSA traits is important not only for abiotic stress tolerance but also for efficient nutrient and water acquisition (Gupta et al., 2017; Griffiths et al., 2022). RSA traits primarily include root length, surface area, and the number of root tips, which influence the major components and spatial arrangement of root systems, significantly affecting water and nutrient uptake (Atkinson et al., 2015; Maccaferri et al., 2016; Liu et al., 2019; Li et al., 2020; Ma et al., 2022; Maqbool et al., 2022).

Previous breeding programs predominantly focused on aboveground traits such as disease resistance, grain quality, and harvest index, while the application of RSA traits has been limited due to the complexity of phenotypic evaluation (Rogers and Benfey, 2015; Roselló et al., 2019; Mehrabi et al., 2020; Rufo et al., 2020; Salarpour et al., 2020; Ober et al., 2021; Pariyar et al., 2021; Saini et al., 2021). Previous studies have indicated that the RSA traits are influenced by environmental factors and controlled by minor genes (Xie et al., 2017; Soriano and Alvaro, 2019; Maqbool et al., 2022). To accelerate the progress of breeding for RSA-related traits, it is imperative to identify the significant associated genomic regions (Soriano and Alvaro, 2019; Yang et al., 2021a). Nowadays, with the advancement of high-throughput genotyping, such as re-sequence and SNP assay (Wang et al., 2014), genome-wide linkage mapping has been widely employed to elucidate the genetic basis of complex traits. Over the past two decades, numerous QTLs have been identified for RSA-related traits (Maccaferri et al., 2016; Xie et al., 2017; International Wheat Genome Sequencing Consortium (IWGSC) et al., 2018; Ye et al., 2018; Soriano and Alvaro, 2019; Yang et al., 2021a; Yang et al., 2021b; Xu et al., 2023).

In this study, we conducted linkage mapping for RSA traits using the wheat 90K assays in a biparental recombinant inbred line (RIL) population derived from the Doumai/Shi 4185 cross. The primary objective of this study is to uncover the genetic basis of these traits and develop available KASP markers for improving wheat RSA.

## Materials and methods

### Plant materials

Doumai is a derivative line of Hesheng 2, which originate from Yuanfeng 6 by Cobalt-60 radiation, whereas Shi 4185 is a widely grown winter wheat cultivar. The 262 F_2:6_ RILs derived from the Doumai/Shi 4185 were used for evaluating RSA-related traits. A hydroponic experiment was conducted with three replicates in a greenhouse. There were 20 seeds from each line that were surface sterilized with10% H_2_O_2_ for 20 min. Subsequently, the cleaned seeds were placed in Petri dishes with moist filter paper. When the coleoptiles reached about 2 cm in length, wheat seedlings were transferred to plastic trays (53 × 27 cm) containing Hoagland’s nutrient solution. Plastic trays were kept in 25°C with a 16-h light and 8-h darkness cycle. After 3 weeks, roots were evaluated for RSA-related traits (**Table S1**). Additionally, a diverse panel of 165 wheat cultivars primarily originating from the Yellow-Huai Wheat Region were also assessed for TRL, TRA, and NRTRAA traits to validate the effectiveness of competitive allele-specific PCR (KASP) markers.

### Phenotype evaluation

Three RSA-related traits, namely, total root length (TRL), total root area (TRA), and number of root tips (NRT), were assessed using the WinRHIZO root analysis system (LA6400XL). The roots were arranged systematically in a dish and scanned using the Expression 11000XL. Subsequently, the images were imported into WinRHIZO software and analyzed using a fixed threshold parameter of 40. Each accession was scored on five plants for RSA traits, and means of three replicates were obtained. Basic statistical analyses and frequency distributions were conducted using SAS v9.3 (http://www.sas.com).

### Linkage map construction

Both RILs and parents were genotyped using the wheat 90K SNP arrays (80,547 SNPs) by CapitalBio Corporation. SNPs with missing data >20% or minor allele frequency (MAF) <0.5 were filtered for further analysis. The filtered SNPs were then analyzed using the BIN function of IciMapping v4.2 (Meng et al., 2015) and grouped into bin markers, which were used to construct a linkage map employing the regression mapping algorithm by JoinMap v4.0. The linkage maps have been reported by Wen et al. (2017) and Li et al. (2018).

### QTL mapping

The inclusive composite interval mapping (ICIM) method using IciMapping v4.1 (Meng et al., 2015) was applied in this study. The logarithm of odds (LOD) threshold for declaring significant QTL was set as 2.62 based on 1000 permutation. The physical positions of SNPs were based on the IWGSC v1.0.

### KASP marker development and validation

SNPs flanking QTL with higher PVE (*QTRL.caas-4A.1*, *QTRA.caas-4A*, and *QNRT.caas-5D*) were converted to KASPs (Rasheed et al., 2016) and designed using PolyMarker (http://www.polymarker.info/) (**Table S2**). The 384-well plates were read on PHERA starplus SNP (BMG Labtech GmbH, Ortenberg, Germany) and the genotype analysis was carried out using KlusterCaller (LGC, Hoddesdon, UK). All the KASPs were validated by 165 cultivars from the Yellow and Huai Wheat Region (Liu et al., 2019).

### Search for candidate genes for RSA-related traits

To identify candidate genes involved in the QTL for RSA-related traits detected in the Doumai/Shi 4185 RIL population, the genes located in the LD block region around the peak SNP ( ± 3.0 Mb kb based on previous LD decay analysis) of each QTL were extracted from the wheat IWGSC v1.1 annotation (https://wheat.pw.usda.gov/GG3/). Genes, excluding hypothetical proteins, transposon proteins, and retrotransposon proteins with SNPs in the coding region, were considered as candidate genes. Quantitative real-time PCR (qRT-PCR) was conducted to test expression differences of the candidate genes between Doumai and Shi 4185. The roots were sampled for RNA extraction after phenotyping. RNA was extracted using the TRIzol method, and the cDNA was synthesized with the HiScript II 1st Strand cDNA Synthesis Kit. Primers were designed using the Primer Premier V5.0. PCR was conducted with a mixture of 20 μl, including 2 μl cDNA, 10 μl ChamQ Universal SYBR qPCR Master Mix, and 0.4 μl of each primer. qRT-PCR was conducted in the ABI StepOnePlus Real-Time PCR System with Tower, and the gene expression level was analyzed by the 2^–ΔΔCT^ method. All assays were performed in two biological replicates and three technical replicates. *TaActin1* was used as the internal control to normalize the expression levels of different samples.

## Results

### Phenotypic evaluation

All three RSA-related traits exhibited continuous and significantly wide variation across the 262 RILs (**Figure S1**). The means of TRL, TRA, and NRT were 74.8 cm (range: 40.2 cm–111.9 cm), 10.4 cm^2^ (range: 6.9 cm^2^–15.2 cm^2^), and 328.2 root tips (range: 94.7–772.0). The standard deviation and coefficient of variation for TRL, TRA, and NRT were 18.1 cm (24.2%), 1.7 cm^2^ (16.4%), and 126.4 (38.5%), respectively. A significant correlation was observed between TRL, TRA, and NRT, with the correlation coefficient of 0.603 (*P* < 0.05) between TRL and TRA, 0.371 (*P* < 0.05) between TRL and NRT, and 0.312 (*P* < 0.05) between TRA and NRT.

### QTL identification

**Figure 1:**
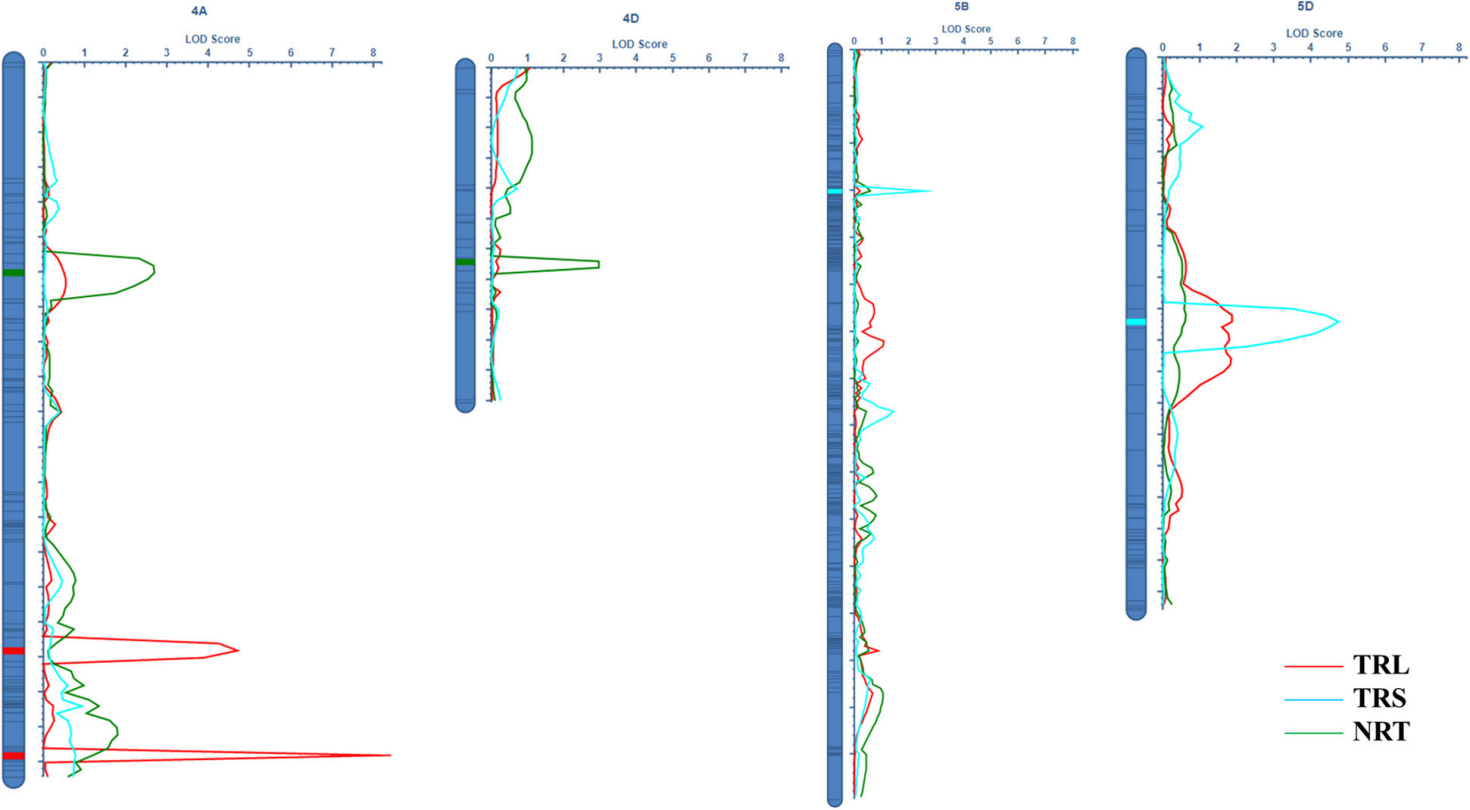
The identified QTLs for RSA related traits in the Doumai × Shi 4185 RIL population.

In total, 11,012 SNP markers were employed for the construction of genetic linkage maps spanning 2030.0 cM (Wen et al., 2017; Li et al., 2018). Two QTLs for TRL were detected on chromosomes 4A, referred to as *QTRL.caas-4A.1* (*BobWhite_c20306_147-Tdurum_contig54973_1510*) and *QTRL.caas-4A.2* (*BS00059454_51-Kukri_c19883_816*), respectively. These QTLs explained 5.94% (additive effect: 5.17) and 9.45% (additive effect: −7.12) of the total phenotypic variances (**Table 1**; **Figure 1**). The favorable allele of *QTRL.caas-4A.1* was contributed by Doumai, while the favorable allele of *QTRL.caas-4A.2* was contributed by Shi 4185. Two QTL for TRA were detected on chromosomes 4A and 4D, named *QTRA.caas-4A* (*BS00041735_51-BS00021715_51*) and *QTRL.caas-4D* (*Ex_c6665_1067-wsnp_J-D_rep_c51623_35119179*), respectively, explaining 7.10% (additive effect: 0.40) and 6.85% (additive effect: −0.39) of the total phenotypic variance. The favorable allele of *QTRA.caas-4A TRA* was contributed by Doumai, while the favorable allele of *QTRA.caas-4D* was originated from Shi4185, respectively. Two QTLs for NRT were detected on chromosomes 5B and 5D and named as *QNRT.caas-5B* (*wsnp_Ex_rep_c67320_65870601-Tdurum_contig10191_996*) and *QNRT.caas-5D* (*IAAV6218-Kukri_c46526_103*), respectively, explaining 5.91% (additive effect: 26.7) and 10.16% (additive effect: 39.1) of the total phenotypic variances. The favorable alleles of *QNRT.caas-5B* and *QNRT.caas-5D* were contributed by Doumai and Shi 4185, respectively (**Table 1**).

**Table 1:** QTL for RSA-related traits in Doumai/Shi 4185 RIL population.

QTL	Genetic interval	Genetic position (cM)	Physical position (Mb)	LOD	R^2^	Add
*QTRL.caas-4A.1*	*BobWhite_c20306_147–Tdurum_contig54973_1510*	84 (82.5–85.5)	702.3–721.3	4.688	5.935	5.174
*QTRL.caas-4A.2*	*BS00059454_51–Kukri_c19883_816*	99 (98.5–99.5)	731.4–732.5	8.204	9.449	−7.120
*QTRA.caas-4A*	*BS00041735_51–BS00021715_51*	30 (27.5–33.5)	594.2–602.9	2.689	7.102	0.402
*QTRA.caas-4D*	*Ex_c6665_1067–wsnp_JD_rep_c51623_35119179*	32 (31.5–33.5)	65.1–69.9	2.958	6.849	−0.392
*QNRT.caas-5B*	*wsnp_Ex_rep_c67320_65870601–Tdurum_contig10191_996*	30 (29.5–30.5)	74.3–78.9	2.565	5.915	26.714
*QNRT.caas-5D*	*IAAV6218–Kukri_c46526_103*	42 (40.5–44.5)	449.6–454.1	4.729	10.157	−39.09

### QTL validation

**Figure 2:**
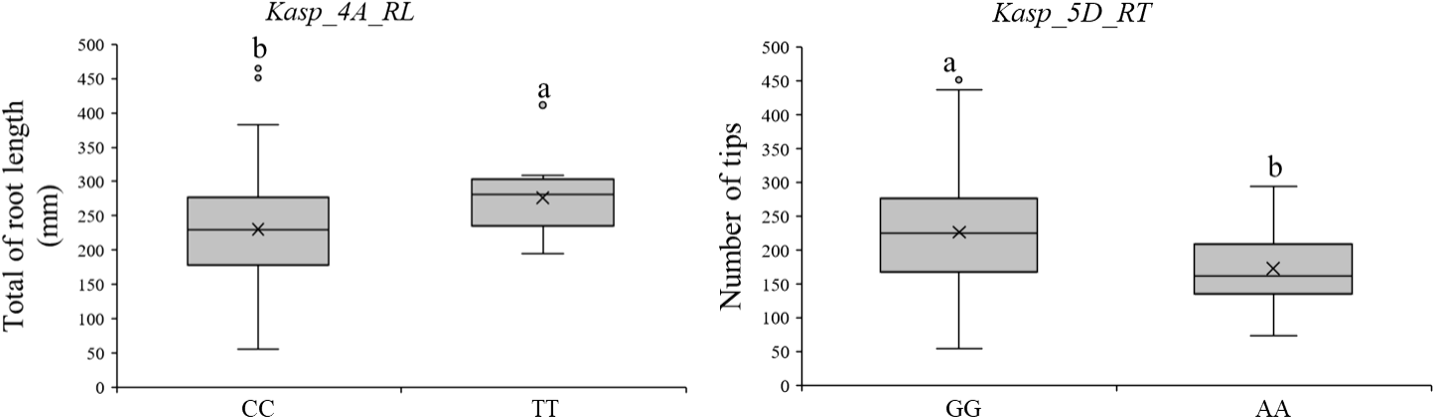
Validating the efficiency of KASP markers for discriminating RSA-related traits. Different lowercase letters means significant different at p<0.05 level.

Three QTL (*QTRL.caas-4A.1, QTRA.caas-4A*, and *QNRT.caas-5D*) with higher phenotypic effects were used to develop KASP markers. Although attempts were made to develop a KASP marker for *QTRA.caas-4A*, it was unable to effectively distinguish between the two parental genotypes in the RIL population. Therefore, the marker did not yield conclusive results. Consequently, two KASP markers, *Kasp_4A_RL* (*QTRL.caas-4A*, *Bobwhite_c20306_147*, 702.3 Mb) and *Kasp_5D_RT* (*QNRT.caas-5D*, *Kukri_c46526_103*, 454.1 Mb), were successfully developed based on the tightly linked SNP markers (**Table 2**; **Figure 2**). A total of 165 diverse cultivars were used to verify the effectiveness of the two KASP markers. For *Kasp_4A_RL*, the favorable allele (TT, account for 75.7%, mean TRL: 276.6 mm) exhibited higher TRL compared to the unfavorable allele (CC, 6.7%, mean TRL: 230.6 mm) at the *P* = 0.05 level (**Table S3**). For *Kasp_5D_RT*, the favorable allele (GG 88.5%, mean NRT: 226.7) showed higher NRT than unfavorable allele (AA, 5.5%, mean NRT: 172.5) at the *P* = 0.05 level (**Table S4**).

**Table 2:** Effects of *Kasp_4A_RL* and *Kasp_5D_RT* on RSA-related traits in the natural population.

QTL	Marker name	Genotype	Number of lines	Phenotype	P-value
*Kasp_4A_RL*	*QTRL.caas-4A*	TT^a^	11	276.6 (TRL)	0.047*
		CC	125	230.5 (TRL)	
*Kasp_5D_RT*	*QNRT.caas-5D*	GG^b^	146	226.7 (NRT)	0.025*
		AA	9	172.5 (NRT)	

^a^TT is a favorable allele; CC is an unfavorable allele.

^b^GG is favorable allele, AA is unfavorable allele.

* Significant at P < 0.05.

### Candidate gene identification

In total, seven candidate genes were selected, primarily involved in the biological metabolism of plant hormones, cellulose, and the ubiquitin pathway (**Table 3**). Among these candidates, *TraesCS4A01G296500* (*QTRA.caas-4A*) encoded an ethylene-regulated nuclear protein (ERT2)-like protein; *TraesCS4A01G436700* (*QTRL.caas-4A.1*) encoded a calcium-binding protein kinase (CDPK)-related kinase; *TraesCS4A01G456300* (*QTRL.caas-4A.1*) encoded a cellulose synthase-like protein; *TraesCS4A01G469400* (*QTRL.caas-4A.2*) encoded an F-box protein; and *TraesCS4D01G092000* (*QTRA.caas-4D*) encoded an ABC transporter, whereas both the *TraesCS5D01G378400* and *TraesCS5D01G38020*0 for *QNRT.caas-5D* encoded E3 ubiquitin-protein ligase. The expressions of the seven candidate genes in Doumai and Shi 4185 were detected using qRT-PCR. Of these, *TraesCS4A01G469400*, *TraesCS4D01G092000*, and *TraesCS5D01G380200* showed no significant differences between the parents, whereas *TraesCS4A01G296500*, *TraesCS4A01G436700*, *TraesCS4A01G456300*, and *TraesCS5D01G378400* showed more than 1.4–3.9-fold higher expression in Doumai compared to Shi4185 (**Figure S2**).

**Table 3:** The candidate genes for RSA-related traits identified in the Doumai/Shi 4185 RIL population.

QTL	Candidate gene	Chromosome	Start (bp)	End (bp)	Annotation
*QTRA.caas-4A*	*TraesCS4A01G296500*	4A	596570488	596571926	Ethylene-regulated nuclear protein (ERT2)-like protein
*QTRL.caas-4A.1*	*TraesCS4A01G436700*	4A	706537515	706538039	Calcium-binding protein kinase (related kinase)
*QTRL.caas-4A.1*	*TraesCS4A01G456300*	4A	721381316	721385942	Cellulose synthase-like protein
*QTRL.caas-4A.1*	*TraesCS4A01G469400*	4A	731097652	731098851	F-box protein
*QTRA.caas-4D*	*TraesCS4D01G092000*	4D	67114234	67118344	ABC transporter G family member
*QNRT.caas-5D*	*TraesCS5D01G378400*	5D	449990773	449992606	E3 ubiquitin-protein ligase
*QNRT.caas-5D*	*TraesCS5D01G380200*	5D	450686195	450688369	E3 ubiquitin-protein ligase

## Discussion

Optimization of crop root systems has long been proposed. However, genetic improvement of crop roots has been rarely attempted (Maccaferri et al., 2016; Yang et al., 2021a). A comprehensive understanding of the genetic basis of RSA traits would facilitate the optimization of root systems under nutrient deficiency (Gu et al., 2016; Xie et al., 2017; Yang et al., 2021a; Yang et al., 2021b). In this study, we identified two QTLs each for TRL (*QTRL.caas-4A.1* and *QTRL.caas-4A.2*), TRA (*QTRA.caas-4A* and *QTRA.caas-4D*), and NRT (*QTRA.caas-5B* and *QTRA.caas-5D*) TRA. Each of these QTLs accounted for 5.94%–9.47%, 6.85%–7.10%, and 5.91%–10.16% of the phenotypic variance, respectively.

Yang et al. (2021b) have identified five QTLs for root length (chromosomes 1A, 2B, 3B, and 7D), three QTLs for root tips (chromosomes 4A, 5A, and 7D), and nine QTLs for root surface (chromosomes 1A, 3A, 4B, and 4D) by linkage mapping in 198 doubled haploid lines of the Yangmai 16/Zhongmai 895 cross. Of the QTLs identified by Yang et al. (2021b), 4A (17.03 Mb–17.05 Mb) and 4D (16.64 Mb–30.66 Mb and 100.69 Mb–108.96 Mb) are different with the loci identified in our study (4A: 594.2 Mb–602.9 Mb, 702.3 Mb–721.3 Mb, and 731.4 Mb–732.5 Mb; 4D: 65.1 Mb–69.9 Mb). Liu et al. (2020) have evaluated 10 RSA-related traits at the seedling stage in 111 F_9_ RIL lines and have identified 19 QTLs mainly distributed on chromosomes 1A, 2B, 2D, 3A, 3B, 3D, 5A, and 5D. Of these, the 5D loci (415.8 Mb–457.6 Mb) overlapped with the*QNRT.caas-5D* (449.4 Mb–454.1 Mb) identified in this study. Alemu et al. (2021) have identified 38 QTLs from 167 historical landraces and 25 modern cultivars by GWAS. Of these, the loci on 4A (*IWB21309*, 17.01 Mb) and 5B (*IWB8808*, 701.5 Mb) for RSA traits were also different with the loci identified in this study (4A: 4A: 594.2 Mb–602.9 Mb, 702.3 Mb–721.3 Mb, and 731.4 Mb–732.5 Mb; 5B: 74.3–78.9). Furthermore, Saini et al. (2021) have identified six meta-QTLs for root-related traits on chromosome 4A by meta-analysis; *MQTL4A.1* (651.78 Mb–705.73 Mb) and *MQTL4A.6* (600.04 Mb–691.14 Mb) significantly associated with root length, root surface, and root tips overlapped with *QTRL.caas-4A.1* (702.3 Mb–721.3 Mb) and *QTRA.caas-4A* (594.2 Mb–602.9 Mb). Xu et al. (2023) also identified 25 QTLs for root and shoot-related traits in 142 RILs derived from Xiaoyan 54 and Jing 411 cross, mainly located on chromosomes 1A, 3A, 4A, and 5B and different with the loci identified in this study. Previous studies have indicated that genes associated with plant height and vernalization may also have certain effects on root system establishment. Furthermore, multiple genes related to plant height and vernalization are found on chromosomes 4D (*Rht2/Rht10* 19.18 Mb, *SVP3-4D/BM1-4D* 469.46 Mb, *Vrn2-4D/ZCCT1-4D* 509.43 Mb), 5B (*TaDEP1-5B* 381.54 Mb, *Vrn1-5B*, 577.00 Mb, *Q-5B* 658.75 Mb), and 5D (*TaDEP1-5D* 329.11 Mb, *Rht23* 524.96 Mb, and *Vrn1-5D* 470.00 Mb) (Xu et al., 2023). Based on physical position, the RSA-related genes identified in this study (*QTRA.caas-4D* 65.1 Mb–69.9 Mb, *QNRT.caas-5B* 74.3 Mb–78.9 Mb, *QNRT.caas-5D* 449.6 Mb–454.1 Mb) are different from the reported wheat genes associated with plant height and vernalization. Therefore, they represent novel genes associated with RSA-related traits. Above all, compared with the previous results and meta-analysis, *QTRL.caas-4A.2*, *QTRL.caas-4D*, *QNRT.caas-5B*, and *QNRT.caas-5D* were novel.

In our study, we conducted linkage mapping for agronomic traits in the Doumai/Shi 4185 RIL population (Li et al., 2018) and identified several regions associated with both RSA-related traits and agronomic traits. Specifically, we found that *QTRL.caas-4A.1* (702.3 Mb–721.3 Mb) co-located influencing QTL clusters related to thousand kernel weight (*QTKW.caas-4AL.1*, 708.6 Mb, *IWB42202*) (Li et al., 2018). Additionally, the loci for RSA traits on chromosomes 5D (*IAAV6218*, 449.6 Mb–454.1 Mb) co-located with regions affecting QTL clusters related to yield, including SN, KNS, TKW, FLW, and KL (*IWB61072-IWB49479*, 382.9 Mb–465.6 Mb) (Li et al., 2018). These results indicated that RSA traits loci could also be targeted to enhance yield potential and stability.

Four genes involved in the biological metabolism of plant hormones, cellulose, and the ubiquitin pathway were identified as high confidence candidate genes. Among these, *TraesCS4A01G436700* of *QTRL.caas-4A.1* encodes the CDPK-related kinase. In plants, CDPKs play a critical role in various signaling pathways and are pivotal in root growth and development (Yip Delormel and Boudsocq, 2019; Dekomah et al., 2022). Silencing CDPK in *Medicago truncatula* led to a significant reduction in root hair growth and cell length (Ivashuta et al., 2005). *TRA* Another candidate gene for *QTRL.caas-4A.1* is *TraesCS4A01G456300*, encoding a cellulose synthase-like protein, which holds importance in plant growth-related and stress-responsive activities (Karas et al., 2021; Lou et al., 2022). *TraesCS4A01G296500*, associated with *QTRA.caas-4AS*, encodes an ethylene-regulated nuclear protein (ERT2)-like protein. Ethylene, a simple small molecule, plays a vital role in signal transduction, cell differentiation, division, and elongation (Qin et al., 2019). Ethylene has also been reported to influence traits related to RSA, leading to root hair and cluster root formation (Xu et al., 2020). Additionally, *TraesCS5D01G380200*, linked to *QNRT.caas-5D*, encodes an E3 ubiquitin-protein ligase, which plays essential roles in plant development (Chen et al., 2018) and is crucial for primary root growth and shoot development (Ramaiah et al., 2022).

Although conventional breeding has contributed to the enhancement of root system, the selection process is time-consuming and less efficient due to the challenges in field measurements of RSA traits (Rasheed et al., 2017). KASP offers a cost-effective, flexible, and highly accurate approach for MAS breeding. In this study, *Kasp_4A_RL* and *Kasp_5D_RT* were successfully developed based on tightly linked SNP markers, proving to be valuable tools for MAS in breeding programs. Additionally, accessions with more favorable alleles, superior RSA traits and appropriate agronomic traits, such as Yumai 18, Jinhe 9123, Yumai 34, Bainong 3217, Lumai 6, Jinmai 61, Lumai 5, Lumai 23, and Yumai 57 are recommended as parental lines for improvement of RSA traits.

## Conclusion

In this study, we identified six QTLs associated with wheat RSA traits and successfully developed two KASP markers that can be utilized in wheat breeding programs aimed at achieving higher and more stable yields. This research serves as a foundational step towards gene cloning and enhancement of wheat root systems.

## Data availability statement

All datasets generated for this study are included in the article or **Supplementary Material**; further inquiries can be directed to the first author.

## Author contributions

YJ: Data curation, Funding acquisition, Writing – original draft. YW: Data curation, Software, Validation, Writing – original draft, Writing – review & editing. JL: Software, Validation, Writing – review & editing. FW: Formal Analysis, Resources, Software, Investigation, Writing – review & editing. XQ: Investigation, Project administration, Writing – review & editing. PL: Funding acquisition, Supervision, Validation, Writing – original draft.

## Supplementary material

Supplementary Figure 1 Frequency distributions for RSA related traits in the Doumai/Shi 4185 RIL population. TRL: total root length; TRA: total root surface area; NRT: number of root tips.

Supplementary Figure 2 qRT-PCR of the seven candidate genes identified in Doumai/Shi 4185 RIL population.
