# Supplementary figures and images for: Genome-wide linkage mapping of root system architecture-related traits in common wheat (Triticum aestivum L.)
Source: Front Plant Sci. 2023 Oct 13;14:1274392. doi: 10.3389/fpls.2023.1274392 (PMC10612324; doi:10.3389/fpls.2023.1274392)

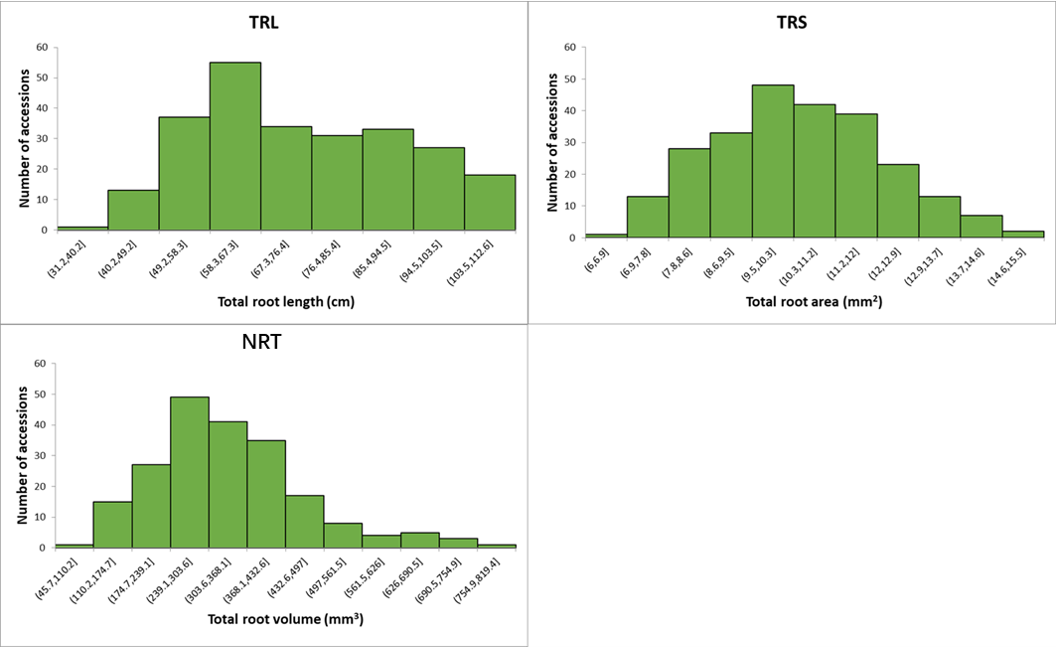

Supplement: Supplementary Figure 1 — Frequency distributions for RSA related traits in the Doumai/Shi 4185 RIL population. TRL: total root length; TRA: total root surface area; NRT: number of root tips. [file Image_1.tif]
