# Supplementary material for: Genome-wide linkage mapping of root system architecture-related traits in common wheat (Triticum aestivum L.)
Source: Front Plant Sci. 2023 Oct 13;14:1274392. doi: 10.3389/fpls.2023.1274392 (PMC10612324; doi:10.3389/fpls.2023.1274392)
Supplement: Supplementary file 4 [file Table_2.docx]

**Table 2** Developed KASP markers for RSA related traits

| **SNP Marker** | **KASP Marker** | **Physical Position (Mb) ^a^** | **Primer Nameb** | **Sequence (5’to 3’) c** |
| --- | --- | --- | --- | --- |
| *Bobwhite_c20306_147* | *Kasp_4A_RL* | 594.2 | FAM | **GAAGGTGACCAAGTTCATGCTACGCCACTCATGCTGGTA** |
|  |  |  | HEX | **GAAGGTCGGAGTCAACGGATTACGCCACTCATGCTGGTG** |
|  |  |  | R | TGATCGCTCTGCCAGTTTAC |
| *Kukri_c46526_103* | *Kasp_5D_RT* | 449.6 | FAM | **GAAGGTGACCAAGTTCATGCTGAGAAGGGATGGAGATAGATCAAT** |
|  |  |  | HEX | **GAAGGTCGGAGTCAACGGATTGAGAAGGGATGGAGATAGATCAAC** |
|  |  |  | R | AGCAGTCTTCTTCTCCCTCG |

^a^ physical position according to the IWGSC V1.0.
