# Supplementary material for: Genome-wide linkage mapping of root system architecture-related traits in common wheat (Triticum aestivum L.)
Source: Front Plant Sci. 2023 Oct 13;14:1274392. doi: 10.3389/fpls.2023.1274392 (PMC10612324; doi:10.3389/fpls.2023.1274392)
Supplement: Supplementary file 7 [file Table_5.docx]

**Table S6.** The primers used for the qRT-PCR of the candidate gene identified in Doumai/Shi 4185 RIL population

| **Candidate gene** | **Primer-F** | **Primer-R** |
| --- | --- | --- |
| *TraesCS4A01G296500* | TACTTGCATCAACCCTGGGAG | GTTCCCCTTGGTAGCTGTGAC |
| *TraesCS4A01G436700* | ATTGCTCTGTGACAAGGCACG | TCTGGACACTCAATGCACACC |
| *TraesCS4A01G456300* | ACCTGTACCCGTTCGCCAAG | GCTTCATCGATCAGGGGAACT |
| *TraesCS4A01G469400* | CTACCTTGGCAGCGTACGATA | AGAAACTACTGCCCATCTGC |
| *TraesCS4D01G092000* | CACTGTTGTAATAGGGCGCA | TGGGACATACAAGCGCAGATC |
| *TraesCS5D01G378400* | GCCTCTTCCTCCTCCATCCT | CAGGAGATGTGGGCTGGAAAT |
| *TraesCS5D01G380200* | CCATGCTGTCGATGGGTATCT | TCTTGACGCTTGAGACTCGAG |
